# Prognostic Value of Lymph Node Necrosis at Different N Stages in Patients with Nasopharyngeal Carcinoma

**DOI:** 10.7150/jca.84854

**Published:** 2023-07-09

**Authors:** Yue-Chun Fu, Shao-Bo Liang, Wen-Jie Huang, Lu-Si Chen, Dan-Ming Chen, Li-Zhi Liu, Min Luo, Xiao-Fen Zhong, Xiang-Ying Xu

**Affiliations:** 1 Department of Laboratory Medicine, The Third Affiliated Hospital of Sun Yat-Sen University, Guangzhou 510630, China.; 2 Department of Radiation Oncology, The Third Affiliated Hospital of Sun Yat-Sen University, Guangzhou 510630, China.; 3 Department of Radiology, Sun Yat-sen University Cancer Center, State Key Laboratory of Oncology in South China, Collaborative Innovation Center for Cancer Medicine, Guangdong Key Laboratory of Nasopharyngeal Carcinoma Diagnosis and Therapy, Guangzhou 510060, China.; 4 Radiotherapy Department of Nasopharyngeal Carcinoma, Cancer Center, The First People's Hospital of Foshan Affiliated to Sun Yat-sen University, Foshan 528000, China.; 5 Department of Molecular Radiation Oncology, German Cancer Research Center, Heidelberg 69120, Germany.; 6 State Key Laboratory of Oncology in South China, Sun Yat-sen University Cancer Center, Collaborative Innovation Center for Cancer Medicine, Guangdong Key Laboratory of Nasopharyngeal Carcinoma Diagnosis and Therapy, Guangzhou 510060, China.; 7 Biotherapy Centre, The Third Affiliated Hospital of Sun Yat-Sen University, Guangzhou 510630, China.

**Keywords:** nasopharyngeal carcinoma, stage N1-3, lymph node necrosis, induction chemotherapy, prognosis

## Abstract

**Background:** Lymph node necrosis (LNN), including retropharyngeal nodal necrosis and cervical nodal necrosis, which is related to radiotherapy/ chemotherapy resistance, is a common phenomenon in nasopharyngeal carcinoma (NPC). This study was to assess the prognostic value of LNN at different N stages in NPC patients.

**Materials and Methods:** In total, 1,665 newly diagnosed NPC patients at stage TxN1-3M0 from two centers were enrolled. Univariate and multivariate models were constructed to assess the association between LNN and long-term survival outcomes. The propensity score matching method was performed to balance treatment groups for baseline characteristics.

**Results:** Of the 1,665, 540 patients (540/1665, 32.4%) were diagnosed with LNN, of which 54.1% (292/540) patients were at stage N1, 31.3% (169/540) at stage N2, and 14.6% (79/540) at stage N3. Univariate and multivariate analyses indicated LNN as an independent predictor for progression‑free survival (PFS), overall survival (OS), distant metastasis-free survival (DMFS), and locoregional relapse-free survival (LRRFS) in stage N1-3 patients (all *P*<0.001). When patients were analyzed according to stage, similar findings were observed for N1 patients (all *P*<0.001); for N2 patients, LNN independently predicted PFS (*P*=0.003), OS (*P*=0.011), and DMFS (*P*=0.004), and for stage N3, LNN only independently predicted LRRFS (*P*=0.019). 123 pairs of patients who received induction chemotherapy plus concurrent chemoradiotherapy or only concurrent chemoradiotherapy were matched, adding induction chemotherapy improved 5-year OS, PFS and LRFFS, but the results were not statistically significant.

**Conclusions:** In NPC patients, LNN could independently predict poor prognosis at all N1-3 stages and at each N stage (N1 to N3). The value of adding induction chemotherapy to concurrent chemoradiotherapy in patients with LNN still requires further prospective studies.

## Introduction

The incidence of nasopharyngeal carcinoma (NPC) has unique geographic distribution, which is rare in the majority of white populations, but particularly prevalent in South China 1-2. Owing to the occult site of primary disease and the highly malignant pathological type, nearly 70% of new cases present with stage III or IV disease in endemic areas 3-4. Moreover, the incidence rate of lymph node metastasis is approximately as high as 80% 5-6.

Lymph node necrosis (LNN), including retropharyngeal nodal necrosis (RNN) and cervical nodal necrosis (CNN), is a common phenomenon of which the incidence rate of CNN is about 30-40% in cervical node metastasis in NPC 7-8. Due to the high incidence of LNN, and the relation between tumor necrosis and radiotherapy/chemotherapy resistance, it is essential to assess how LNN influences the prognosis of patients with NPC. In the early studies by Chua et al., nodal necrosis did not affect nodal response to chemotherapy and radiotherapy, LNN was not an independent factor in predicting treatment outcomes in NPC 9. The following report by Mao et al., also indicated that nodal necrosis was not an independent prognostic factor for NPC 10.

However, in the recent years, Lan et al. found that CNN was a significant independent adverse prognostic factor for overall survival (OS), disease-free survival (DFS), regional relapse-free survival (RRFS), and distant metastasis-free survival (DMFS) in NPC 11. Liu et al. also confirmed that CNN could reliably predict survival risk in NPC patients based on large samples from a multicenter study 8. The previous studies only focus on CNN, not including RNN, which may be because it is difficult to distinguish it from the primary tumor sometimes. However, the incidence rate of RNN is about 20% in NPC, so it is necessary to include RNN in the prognostic analysis 12-13. And there are few reports on the prognostic value of LNN at different N stages in NPC patients.

Furthermore, if LNN is a signal of poor survival in NPC, patients with LNN might require timely strengthening treatment. In the study by Lan *et al*., adding induction chemotherapy to concurrent chemoradiotherapy could achieve better survival in NPC patients with CNN by reducing the risk of death, tumor progression, and distant metastasis 14. In the study by Li *et al*., induction chemotherapy plus concurrent chemoradiotherapy could improve OS in NPC patients with multiple CNNs, compared to concurrent chemoradiotherapy (76.1% *vs.* 55.7%, adjusted *p*=0.030) 15. However, there are few reports on the prognostic benefit of adding induction chemotherapy to concurrent chemoradiotherapy in NPC patients with LNN including both RNN and LNN.

Based on the above controversies, in this study, we aimed to evaluate if LNN including RNN and CNN, can predict survival outcomes at different N stages on large samples from two centers, in order to provide evidence to verify the prognostic value of LNN at each N stage and to make individualized treatment regimen to NPC patients.

## Material*s* and Methods

### Patients

The Ethics Committees of the Third Affiliated Hospital of Sun Yat-Sen University approved this retrospective study, and the need for informed consent was waived from all patients. The authenticity of this article has been validated by uploading the key raw data onto the Research Data Deposit platform (https://rdd.sysu.edu.cn/Guideline/). In this study, 1,665 patients with newly diagnosed, histologically proven, and non-metastatic NPC, who were treated at Sun Yat-sen University Cancer Center (1,042 patients) or First People's Hospital of Foshan Affiliated to Sun Yat-sen University (623 patients) from January 2010 to March 2014 were enrolled. All patients included 1229 men and 436 women (male:female ratio, 2.8:1), with a median age of 46 years (range, 11-83 years).

Some examination relative to the clinical stage was offered before treatment, including nasal endoscopy, magnetic resonance imaging (MRI) of the nasopharynx and neck, chest radiography, abdominal ultrasound, bone scan, and so on. All the patients were restaged according to the 8th edition of the American Joint Commission on Cancer staging system (AJCC) 16. The stage distribution was as follows: 367/1,665 (22%) with T1, 226 (13.6%) with T2, 651 (39.1%) with T3, and 421 (25.3%) with T4; 1162 (69.8%) with N1, 353 (21.2%) with N2, and 150 (9%) with N3; and 427 (25.6%) with stage II, 696 (41.8%) with stage III, and 542 (32.6%) with stage IVa.

### Imaging

All patients underwent MRI of the nasopharynx and neck with a 1.5-T or 3.0-T MRI system before treatment. Detailed protocol of the MRI scan has been previously described 17-18. Two radiologists with >10 years' experience in head and neck carcinomas evaluated the MRI scans independently, and any disagreements were resolved by consensus every two weeks. Metastatic lymph nodes were diagnosed according to the criteria recommended by Van *et al*. and Huang *et al*. 19-20. LNN including RNN and CNN, was diagnosed based on the following criteria: (1) focal area of high signal intensity on T2-weighted images; (2) focal area of low signal intensity on contrast-enhanced T1-weighted images; and (3) with or without a surrounding rim of enhancement 21-22 (Supplementary Figure 1).

### Treatment

All patients were treated using intensity-modulated radiation therapy (IMRT) five fractions per week for 6-7 weeks. The prescribed doses were 66-72Gy in 30-33 fractions to planning target volume (PTV) of primary gross tumor and adjacent metastatic retropharyngeal lymph nodes, and 64-70 Gy in 30-33 fractions to PTV of metastatic cervical lymph nodes, 60-63 Gy in 30-33 fractions to PTV of high-risk clinical target volume (CTV1), and 50-56 Gy in 28-33 fractions to PTV of low-risk CTV (CTV2). Boost irradiation not exceeding 16Gy, was proposed to the patients with obvious residual disease in the primary tumor or metastatic lymph nodes at the end of IMRT. The detailed protocol has been described in our previous reports 23-24.

Of the 1,665, 1,560 patients (93.7%) received platinum-based chemotherapy based on the treatment principles of NPC at the two institutes 12, 25. Of these, 961 received induction chemotherapy plus concurrent chemotherapy, of which induction chemotherapy was composed of 2-3 cycles of PF (cisplatin 80 mg/m^2^, day1 + fluorouracil 1000 mg/m^2^/d, days 1-4 or 800 mg/m^2^/d, days 1-5), TPF (docetaxel 60 mg/m^2^, day1 + cisplatin 60 mg/m^2^, day1 + fluorouracil 600 mg/m^2^/d, days 1-5), TP (docetaxel 75-80 mg/m^2^ + cisplatin 75-80 mg/m^2^, day1) or GP (gemcitabine 1000 mg/m^2^/d, days 1, 8 + cisplatin 80 mg/m^2^, day1) regimens every 3 weeks, and concurrent chemotherapy included cisplatin (40 mg/m^2^) weekly or cisplatin (80-100 mg/m^2^) every 3 weeks; 524 received concurrent chemotherapy, and 75 received induction chemotherapy. In the event of tumor relapse or persistent disease, salvage treatment such as surgery, radiotherapy, or chemotherapy was administered when appropriate.

### Follow-up and statistical analysis

The follow-up time was calculated from the date of starting treatment to the date of last follow-up or the date of event. Patients were followed up every 3 months during the first two years after IMRT and every 6 months thereafter. The main endpoint was progression-free survival (PFS), and the secondary endpoints included OS, DMFS, and locoregional relapse-free survival (LRRFS).

All analyses were performed using SPSS 22.0 (IBM Corp, Armonk, NY, USA). Chi-square or Fisher exact test was used to compare categorical data. Univariate analyses were conducted using the Kaplan-Meier method. Multivariable analyses were performed using the Cox proportional hazards model, in which the following parameters were included: age (≤45 years vs. >45 years), gender (male vs. female), pathological subtype (type 1/2 vs. type 3), T category (T1-2 vs. T3-4), N category (N1 vs. N2-3), chemotherapy (yes vs. no), and LNN (yes vs. no). A propensity score matching method was performed to match patients with LNN receiving induction chemotherapy plus concurrent chemoradiotherapy with those receiving concurrent chemoradiotherapy on a 1:1 basis. Two-sided* p* values < 0.05 were considered statistically significant.

## Results

### Prognostic value of lymph node necrosis on patients at stage N1-3

Of the total 1,665 NPC patients recruited, 540 (540/1665, 32.4%) had LNN, and 1,125 did not show such findings on MRI. Of these 540, 54.1% (292/540) patients were at stage N1, 31.3% (169/540) at stage N2, and 14.6% (79/540) at stage N3. The sociodemographic and clinical characteristics of NPC patients at stages N1-3 with and without LNN were shown in Table 1. The median follow-up time was 67 months (range, 1-122 months).

**Figure 1:**
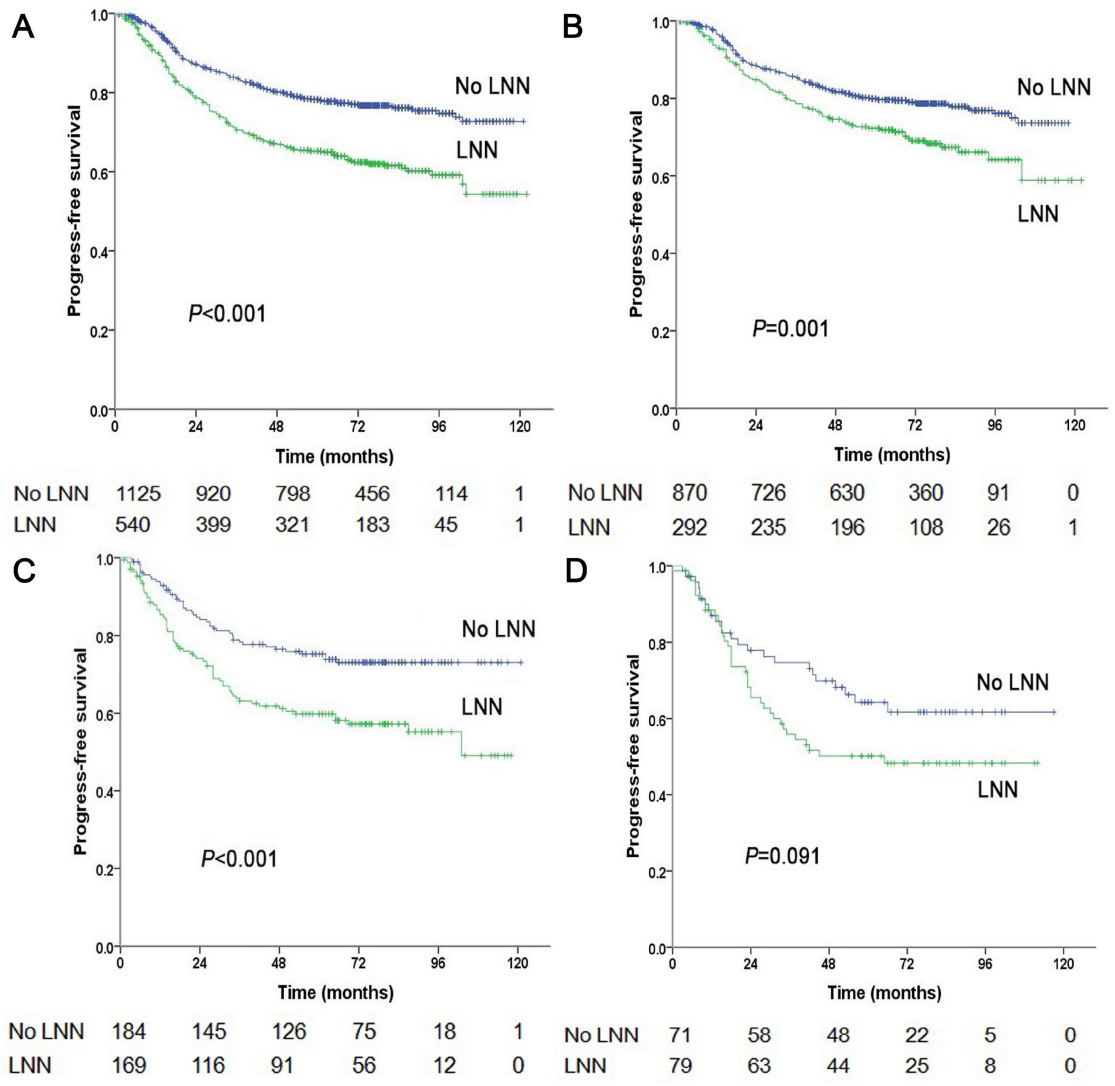
Progression-free survival of NPC patients with and without LNN. (A) 1,665 patients at stage N1-3, (B) 1,162 patients at stage N1, (C) 353 patients at stage N2, (D) 150 patients at stage N3.

**Figure 2:**
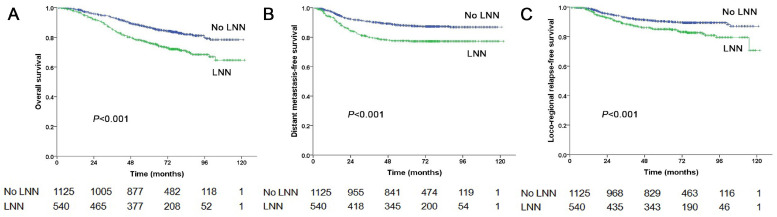
Prognostic comparison of 1,665 stage N1-3 NPC patients with and without LNN. (A) Overall survival, (B) Distant metastasis-free survival, (C) Locoregional relapse-free survival.

The estimated 5-year PFS was 65.2% in patients with LNN compared with 78.2% in those without (hazard ratio [HR]: 1.810, 95% confidence interval [CI]: 1.500-2.183, *P*<0.001, Figure 1A). Similarly, OS was 76% vs. 85% (HR: 1.842, 95% CI: 1.472-2.303, *P*<0.001, Figure 2A); DMFS was 77.5% vs. 88% (HR: 1.954, 95% CI: 1.520-2.511, *P*<0.001, Figure 2B); and LRRFS was 85% vs. 90.4% (HR: 1.714, 95% CI: 1.281-2.293, *P*<0.001, Figure 2C), respectively. Multivariate analyses indicated that in patients at stage N1-3, LNN was an independent predictor of PFS, OS, DMFS, and LRRFS (all *P*<0.001, Table 2).

### Prognostic value of lymph node necrosis on patients at stage N1

**Figure 3:**
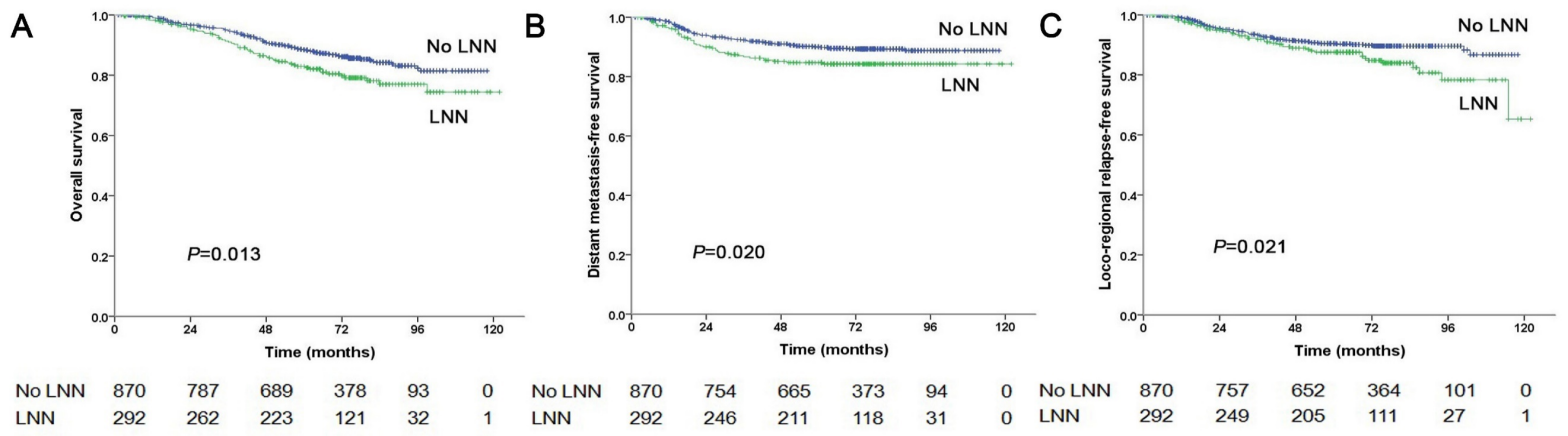
Prognostic comparison of 1,162 stage N1 NPC patients with and without LNN. (A) Overall survival, (B) Distant metastasis-free survival, (C) Locoregional relapse-free survival.

In total, 1,162 patients were at stage N1, of these, 292 patients (292/1162, 25.1%) had LNN and 870 patients did not. The estimated 5-year PFS was 72.3% in patients with LNN compared with 79.9% in those without (HR: 1.544, 95% CI: 1.196-1.994, *P*=0.001, Figure 1B). Similarly, OS was 82.9% vs. 88.5% (HR: 1.502, 95% CI: 1.091-2.069, *P*=0.013, Figure 3A); DMFS was 84.7% vs. 90% (HR: 1.548, 95% CI: 1.073-2.234, *P*=0.020, Figure 3B); and LRRFS was 87.6% vs. 90.4% (HR: 1.556, 95% CI: 1.069-2.266, *P*=0.021, Figure 3C), respectively. Multivariate analyses indicated that in patients at stage N1, LNN was an independent predictor of OS, PFS, DMFS, and LRRFS (all *P*<0.001, Supplementary Table 1).

### Prognostic value of lymph node necrosis on patients at stage N2

**Figure 4:**
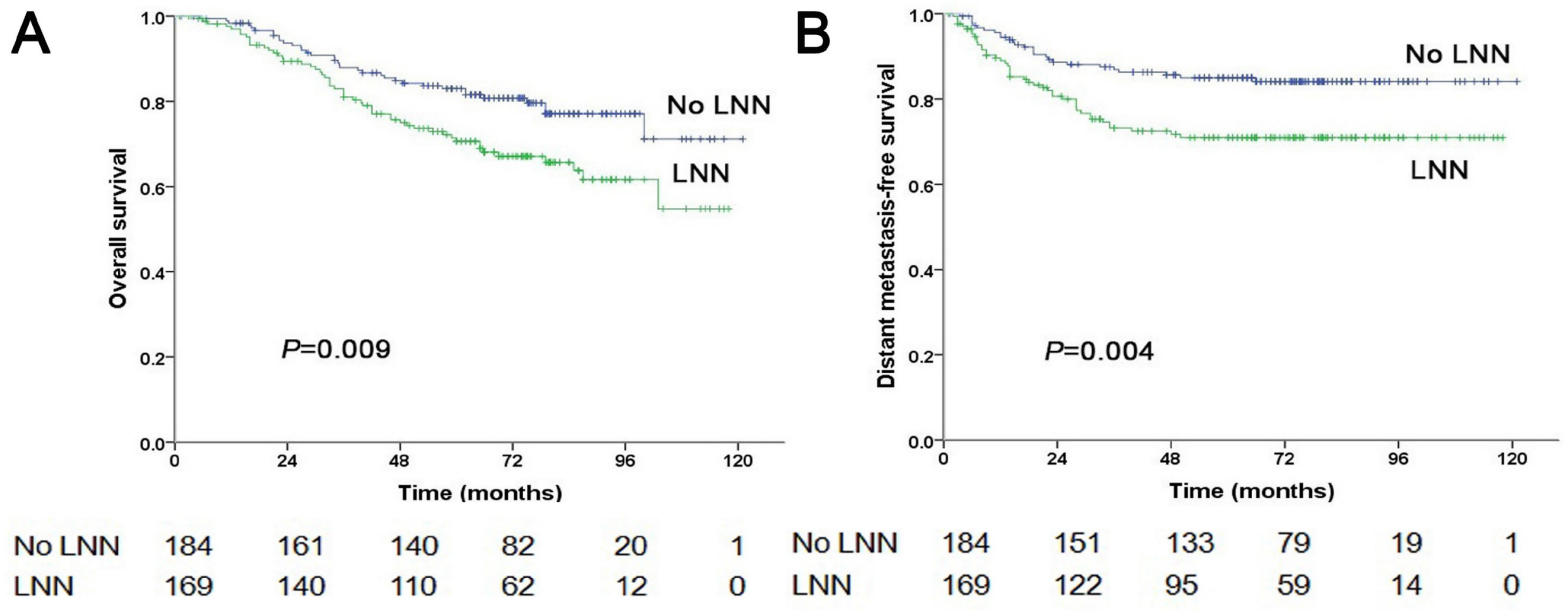
Prognostic comparison of 353 stage N2 NPC patients with and without LNN. (A) Overall survival, (B) Distant metastasis-free survival.

Of the 353 patients at stage N2, 169 patients (169/353, 47.9%) had LNN and 184 did not. The estimated 5-year PFS was 59.8% in patients with LNN compared to 75.3% in those without (HR: 1.861, 95% CI: 1.281-2.703, *P*=0.001, Figure 1C). Similarly, OS was 70.6% vs. 83% (HR: 1.754, 95% CI: 1.148-2.679, *P*=0.009, Figure 4A); DMFS was 70.9% vs. 85% (HR: 2.032, 95% CI: 1.261-3.275, *P*=0.004, Figure 4B); and LRRFS was 83.4% vs. 88.4% (*P*=0.251), respectively. Multivariate analyses indicated that in patients at stage N2, LNN was an independent predictor of OS (*P*=0.011), PFS (*P*=0.003), and DMFS (*P*=0.004; Supplementary Table 2).

### Prognostic value of lymph node necrosis on patients at stage N3

Of the 150 patients at stage N3, 79 patients (79/150, 52.7%) had LNN and 71 did not. The estimated 5-year PFS was 50.2% for patients with LNN compared to 64.3% for patients without (HR: 1.556, 95% CI: 0.932-2.595, *P*=0.091, Figure 1D). The OS was 62.8% vs. 73.3% (*P*=0.191); the DMFS was 64.1% vs. 69.5% (*P*=0.408); and the LRRFS was 78.3% vs. 94.8% (HR: 3.746, 95% CI: 1.242-11.298, *P*=0.019, Supplementary Figure 2), respectively. Multivariate analyses indicated that in patients at stage N3, LNN was an independent predictor of LRRFS (*P*=0.019).

### Prognostic value of induction chemotherapy on patients with lymph node necrosis

Propensity score-matching was performed for patients with LNN. Of the total, 246 patients were matched, including 123 patients who received induction chemotherapy plus concurrent chemoradiotherapy and 123 patients who only received concurrent chemoradiotherapy. Between the two groups, age, gender, pathological subtype, T stage, N stage, and clinical stage were not significantly different (Supplementary Table 3). The estimated 5-year PFS was 74.1% in patients who received induction chemotherapy compared to 66.6% in those who did not (*P*=0.348). OS was 86.0% vs. 78.9% (*P*=0.310); DMFS was 82.6% vs. 81.7% (*P*=0.910); and LRRFS was 90.2% vs. 84.1% (*P*=0.289), respectively. Adding induction chemotherapy to concurrent chemoradiotherapy improved 5-year PFS, OS and LRFFS, but the results were not statistically significant.

## Discussion

In the present study, the incidence rates of LNN in NPC patients at stage N1-3, stage N1, stage N2 and stage N3 were 32.4%, 25.1%, 47.9%, and 52.7%, respectively. The patients with LNN had poorer survival than those without at all N1-3 stages and at each N stage (N1 to N3). Induction chemotherapy followed by concurrent chemoradiotherapy improved the 5-year PFS, OS and LRFFS rates compared to concurrent chemoradiotherapy alone, but the results were not statistically significant.

The incidence rate of LNN in stage N1-3 NPC patients was 32.4% in this study, similar to 23-42% reported in previous studies 8, 26-27. This study further evaluated the incidence of LNN at different N stages, that was 25.1% with N1, 47.9% with N2, and 52.7% with N3. The incidence rates of LNN at stage N2 or N3 were significantly higher than that at stage N1. In the other study by Bin *et al*., the ratio of tumor necrosis in cervical nodes ranged from 0.4% to 78.49% 28. Besides NPC, lymph nodes with poor blood supply, are prone to occur necrosis because of tumor hypoxia, which also has high incidence rate in other carcinamas 29.

In the present study, LNN was an independent predictor of poor prognosis in NPC patients with lymph node metastasis. This result was similar to that in previous reports, in which CNN was also a poor prognostic factor 8, 11. And the prognosis would become worse when the ratio of nodal necrosis increased 29. Our previous study has confirmed that tumor necrosis resulted in residual tumor at the end of treatment, and ultimately affected long-term survival 12. The possible reasons could be that the tumor was resistant to radiotherapy and chemotherapy in hypoxic microenvironment. As reported in fundamental researches, hypoxic microenvironment would stimulate cancer cells to produce hypoxia inducible factor (HIF), which could reduce cell proliferation, reactive oxygen species and DNA damage, leading to radiation resistance 30-31. The hypoxia could induce epithelia-mesenchymal transition (EMT), which is also an important mechanism of radiation resistance 32-33.

This study further confirmed the negative prognostic impact of LNN at each N stage (N1 to N3), respectively. Univariate and multivariate analyses indicated LNN as an independent predictor for PFS, OS, DMFS, and LRRFS for N1 patients; for N2 patients, LNN independently predicted PFS, OS, and DMFS, and for stage N3, LNN independently predicted LRRFS. The different results of subgroup analysis of N1-3 could be due to tumor heterogeneity. In the other study by Feng *et al*., patients of stage N1 with LNN were proposed to reclassify as stage N2, and stage N2 with LNN as stage N3 33. Due to the poor prognosis of NPC patients with LNN, it is necessary to explore some intensive treatment for them.

However, it was still unknown what was the suitable treatment for the NPC patients with LNN. In the present study, adding induction chemotherapy to concurrent chemoradiotherapy improved survival in patients with LNN, but the results were not statistically significant. In studies by Li *et al*., and Lan *et al*., NPC patients with cervical nodal necrosis who received induction chemotherapy followed by concurrent chemoradiotherapy showed better survival than those who received concurrent chemoradiotherapy alone 14-15. Nevertheless, the value of induction chemotherapy in patients with LNN still requires further prospective studies.

To our knowledge, this study is the first to confirm the prognostic value of LNN at different N stages in NPC patients by univariate and multivariate analyses based on a large sample from two centers. However, our study has some limitations. First, due to the nature of retrospective studies, chemotherapy regimens were inconsistent which might affect the survival of patients. Second, the IMRT regimens for NPC might have been slightly different in the two centers; despite this, the treatment outcome was similar in the two centers, according to the previous reports 23-24.

## Conclusions

In summary, LNN is common in NPC patients at stage N1-3, and its incidence gradually increases as N stage advances. LNN was an independent predictor of poor prognosis in NPC patients at all N1-3 stages and at each N stage (N1 to N3). The value of adding induction chemotherapy to concurrent chemoradiotherapy in patients with LNN still requires further prospective studies.

## Supplementary Material

Supplementary figures and tables.

## Figures and Tables

**Table 1:** Sociodemographic and clinical characteristics of the participants

Variables	Numbers	PFS			OS			DMFS			LRRFS		
	N=1665 (100%)	surv.	*P* value		surv.	*P* value		surv.	*P* value		surv.	*P* value	
Sex (%)			0.304			0.002			0.170			0.369	
Male	1229 (73.8%)	72.8%			79.7%			84.6%			89.4%		
Female	436 (26.2%)	75%			86.2%			87.2%			87.4%		
Age (years)			0.026			<0.001			0.562			0.355	
≤45	860 (51.7%)	71%			77.3%			85%			88.4%		
>45	805 (48.3%)	75.9%			85.7%			85.6%			89.4%		
Pathological subtype			0.014			0.052			0.126			0,182	
WHO type 1/2	45 (2.7%)	60%			73.3%			77.8%			84.4%		
WHO type 3	1620 (97.3%)	73.8%			81.6%			85.5%			89%		
T category (%)			<0.001			<0.001			<0.001			<0.001	
T1-2	593 (35.6%)	80.6%			87.9%			89.5%			92.1%		
T3-4	1072 (64.4%)	69.4%			77.8%			82.9%			87.1%		
N category (%)			<0.001			<0.001			<0.001			0.070	
N1	1162 (69.8%)	77.1%			85.4%			89%			89.5%		
N2-3	503 (30.2%)	64.8%			72.2%			76.7%			87.5%		
Clinical category (%)			<0.001			<0.001			<0.001			0.001	
II	427 (25.6%)	84.8%			92%			93.4%			92.5%		
III-IVA	1238 (74.4%)	69.5%			77.7%			82.5%			87.6%		
Chemotherapy			0.209			0.054			0.301			0.759	
Yes	1520 (91.3%)	73.8%			81.8%			84.9%			88.9%		
No	145 (8.7%)	69.7%			76.6%			89%			89%		
Lymph node necrosis			<0.001			<0.001			<0.001			<0.001	
Yes	1125 (67.6%)	63.9%			74.1%			78.9%			85.2%		
No	540 (32.4%)	78%			84.9%			88.4%			90.7%		
													

Note 1: *P*-values were calculated using a Cox proportional hazards model. Abbreviations: WHO, World Health Organization; surv, survival.

**Table 2:** Significant factors included in the multivariate analyses for 1,665 nasopharyngeal carcinoma patients at stage N1-3

Endpoint	Variable	HR	95% CI	*P* Value
PFS	Age	1.210	1.001-1.463	0.049
	Pathological subtype	2.000	1.245-3.214	0.004
	T category	1.808	1.460-2.238	<0.001
	N category	1.525	1.252-1.857	<0.001
	Lymph node necrosis	1.643	1.353-1.994	<0.001
	Chemotherapy	1.314	0.959-1.800	0.089
OS	Sex	1.473	1.111-1.955	0.007
	Age	1.584	1.255-1.999	<0.001
	Pathological subtype	1.973	1.104-3.529	0.022
	T category	2.079	1.595-2.711	<0.001
	N category	1.794	1.421-2.265	<0.001
	Lymph node necrosis	1.609	1.275-2.030	<0.001
	Chemotherapy	1.520	1.060-2.180	0.023
DMFS	Pathological subtype	1.743	0.925-3.286	0.086
	T category	1.764	1.322-2.355	<0.001
	N category	2.052	1.583-2.661	<0.001
	Lymph node necrosis	1.634	1.260-2.118	<0.001
LRRFS	Pathological subtype	1.896	0.889-4.046	0.098
	T category	1.859	1.334-2.591	<0.001
	Lymph node necrosis	1.706	1.275-2.283	<0.001

Note 1: Hazard ratios and *P*-values were calculated by using the Cox proportional hazards model. Abbreviations: HR, hazard ratio; CI, confidence interval; PFS, progress-free survival; OS, overall survival; DMFS, distant metastasis-free survival; LRRFS, locoregional relapse-free survival.

## Abbreviations

LNN: lymph node necrosis
NPC: nasopharyngeal carcinoma
PFS: progression‑free survival
OS: overall survival
DMFS: distant metastasis-free survival
LRRFS: locoregional relapse-free survival
CNN: cervical nodal necrosis
DFS: disease-free survival
RRFS: regional relapse-free survival
RNN: retropharyngeal nodal necrosis
AJCC: the American Joint Commission on Cancer staging system
MRI: magnetic resonance imaging
IMRT: intensity-modulated radiation therapy
PTV: planning target volume
CTV: clinical target volume
