# Supplementary material for: Prognostic Value of Lymph Node Necrosis at Different N Stages in Patients with Nasopharyngeal Carcinoma
Source: J Cancer. 2023 Jul 9;14(11):2085–92. doi: 10.7150/jca.84854 (PMC10367925; doi:10.7150/jca.84854)
Supplement: Supplementary file 1 — Supplementary figures and tables. [file jcav14p2085s1.pdf]

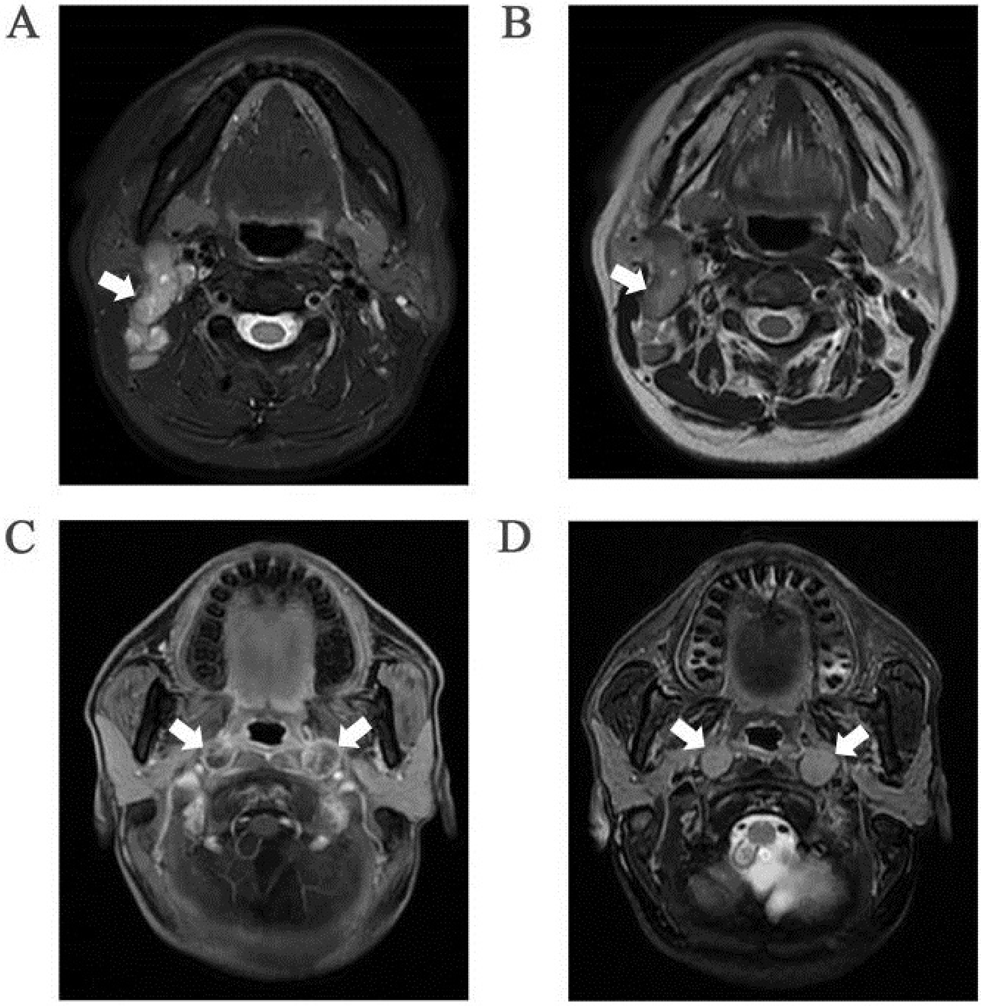

Supplementary Figure 1. Magnetic resonance imaging of patients with NPC shows CNN and RNN. A/B. Contrast-enhanced T1-weighted image/axial T2-weighted image with necrotic lymph nodes in the right level II area (arrows). C/D. Contrast-enhanced T1-weighted image/axial T2-weighted image with necrotic lymph nodes in both retropharyngeal area (arrow).

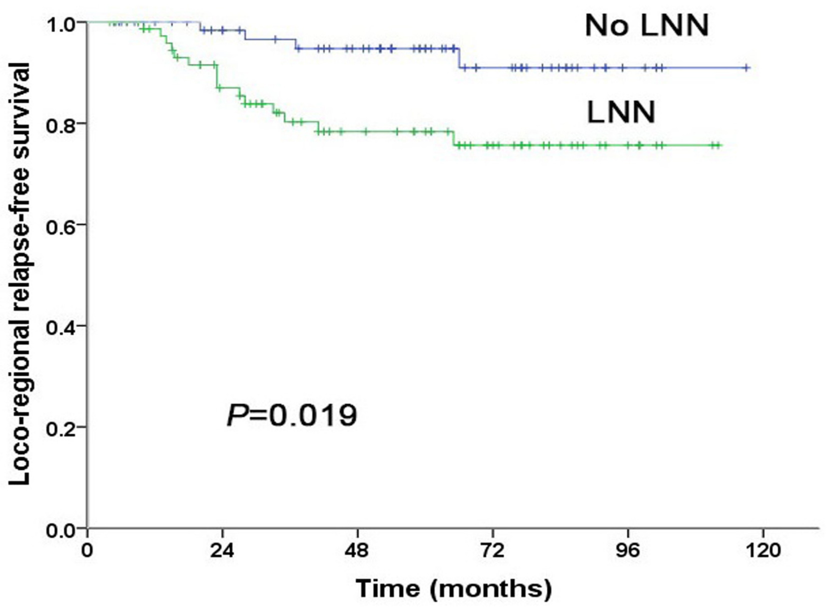

No LNN	71	56	46	21	5	0
LNN	79	56	37	21	7	0

Supplementary Figure 2. Loco-regional relapse-free survival. of 150 stage N3 NPC patients with and without LNN.

Supplementary Table 1: Significant factors included in the multivariate analyses for 1,162 nasopharyngeal carcinoma patients at stage N1

**Endpoint**	**Variable**	**HR**	**95% CI**	***P* Value** [a]
PFS	Age	1.245	1.032-1.503	0.022
	Pathological subtype [b]	2.103	1.309-3.379	0.002
	T category [c]	1.825	1.475-2.259	<0.001
	Lymph node necrosis	1.816	1.505-2.192	<0.001
OS	Sex	1.537	1.159-2.038	0.003
	Age	1.616	1.281-2.038	<0.001
	Pathological subtype [b]	2.112	1.181-3.775	0.012
	T category [c]	2.151	1.649-2.805	<0.001
	Lymph node necrosis	1.868	1.1493-2.337	<0.001
	Chemotherapy	1.479	1.032-2.120	0.033
DMFS	Pathological subtype [b]	1.815	0.963-3.423	0.065
	T category [c]	1.829	1.370-2.441	<0.001
	Lymph node necrosis	1.946	1.513-2.501	<0.001
LRRFS	Pathological subtype [b]	1.896	0.889-4.046	0.098
	T category [c]	1.859	1.334-2.591	<0.001
	Lymph node necrosis	1.706	1.275-2.283	<0.001

Note 1: Hazard ratios and *P*-values were calculated by using the Cox proportional hazards model.
Abbreviations: HR, hazard ratio; CI, confidence interval; PFS, progress-free survival; OS, overall survival; DMFS, distant metastasis-free survival; LRRFS, locoregional relapse-free survival.

Supplementary Table 2: Significant factors included in the multivariate analyses for 353 nasopharyngeal carcinoma patients at stage N2

Endpoint	Variable	HR	95% CI	*P* Value
PFS	Pathological subtype	2.397	0.963-5.966	0.060
	T category	1.463	0.942-2.272	0.090
	Lymph node necrosis	1.765	1.212-2.571	0.003
OS	Age	1.699	1.090-2.647	0.019
	Pathological subtype	2.881	1.029-8.067	0.044
	Lymph node necrosis	1.733	1.135-2.648	0.011
DMFS	Pathological subtype	2.566	0.934-7.044	0.067
	Lymph node necrosis	2.005	1.243-3.232	0.004

Note 1: Hazard ratios and *P*-values were calculated by using the Cox proportional hazards model.
Abbreviations: HR, hazard ratio; CI, confidence interval; PFS, progress-free survival; OS, overall survival; DMFS, distant metastasis-free survival.

Supplementary Table 3: Baseline characteristics of nasopharyngeal carcinoma patients with lymph node necrosis treated with concurrent chemoradiotherapy with and without induction chemotherapy, before and after propensity score matching

	Before PSM				After PSM			
	With induction chemotherapy N=346	Without induction chemotherapy N=126	SMD	*P* Value	With induction chemotherapy N=123	Without induction chemotherapy N=123	SMD	*P* Value
Sex (%)			0.106	0.363			0.018	0.890
Male	258 (74.6%)	88 (69.8%)			85 (69.1%)	86 (69.9%)		
Female	88 (25.4%)	38 (30.2%)			38 (30.9%)	37 (30.1%)		
Age (years)			0.111	0.334			0.065	0.610
≤45	184 (53.2%)	60 (47.6%)			61 (49.6%)	65 (52.8%)		
>45	162 (46.8%)	66 (52.4%)			62 (50.4%)	58 (47.2%)		
Pathological subtype			0.070	0.753			0.182	0.248
WHO type 1	1 (0.3%)	1 (0.8%)			1 (0.8%)	1 (0.8%)		
WHO type 2	6 (1.7%)	2 (1.6%)			0 (0)	2 (1.6%)		
WHO type 3	339 (98%)	123 (97.6%)			122 (99.2%)	120 (97.6%)		
T-stages (%)			0.429	<0.001			0.093	0.913
T1	47 (13.6%)	32 (25.4%)			31 (25.2%)	29 (23.6%)		
T2	50 (14.5%)	27 (21.4%)			30 (24.4%)	27 (22%)		
T3	141 (40.8%)	44 (34.9%)			39 (31.7%)	44 (35.8%)		
T4	108 (31.2%)	23 (18.3%)			23 (18.7%)	23 (18.7%)		
N-stages (%)			0.445	<0.001			<0.001	1.000
N1	173 (50%)	81 (64.3%)			78 (63.4%)	78 (63.4%)		
N2	106 (30.6%)	38 (30.2%)			38 (30.9%)	38 (30.9%)		
N3	67 (19.4%)	7 (5.6%)			7 (5.7%)	7(5.7%)		
Clinical stages (%)			0.638	<0.001			<0.001	1.000
II	46 (13.3%)	47 (37.3%)			44 (35.8%)	44 (35.8%)		
III	141 (40.8%)	49 (38.9%)			49 (39.8%)	49 (39.8%)		
IVA	159 (46%)	30 (23.8%)			30 (24.4%)	30 (24.4%)		

Note 1: *P*-values were calculated using a Cox proportional hazards model.
Abbreviations: WHO, World Health Organization.
